# Correction: Disulfiram as an anti-inflammatory agent: mechanisms, nano-delivery strategies, and applications in non-oncologic diseases

**DOI:** 10.1039/d6ra90023b

**Published:** 2026-02-19

**Authors:** Qiwen Jiang, Mengni Jiang, Yanwei Lv, Xinyuan Zhang, Shige Wang, Jiulong Zhao

**Affiliations:** a Department of Gastroenterology, Shanghai Institute of Pancreatic Diseases, Changhai Hospital, National Key Laboratory of Immunity and Inflammation, Naval Medical University Shanghai China jlzhao9@163.com; b School of Materials and Chemistry, University of Shanghai for Science and Technology No. 516 Jungong Road Shanghai 200093 China sgwang@usst.edu.cn

## Abstract

Correction for ‘Disulfiram as an anti-inflammatory agent: mechanisms, nano-delivery strategies, and applications in non-oncologic diseases’ by Qiwen Jiang *et al.*, *RSC Adv.*, 2025, **15**, 36344–36364, https://doi.org/10.1039/D5RA04662A.

The authors regret that the work of Cvek *et al.* published in *ACS Medicinal Chemistry Letters* was incorrectly cited in ref. 167. The citation was erroneously inserted during referencing and unfortunately overlooked in revision. The citation has now been corrected to:

J. Lanz, N. Biniaz‑Harris, M. Kuvaldina, S. Jain, K. Lewis, B. A. Fallon, *Antibiotics*, 2023, **12**, 524.

The authors initially described the work of Liu *et al.*, published in *Nature Immunology* in 2020 (ref. 12), as “a groundbreaking discovery”. However, subsequent in-depth research has indicated that the antiseptic effect of DSF *in vivo* may be significantly influenced by copper, and their work did not prove that gasdermin D is inhibited *in vivo* by DSF specifically. Moreover, while DSF has been shown to effectively inhibit GSDMD pore formation *in vitro*, its *in vivo* efficacy in blocking this process constitutes an unresolved question. Therefore, the description “a groundbreaking discovery” may not be appropriate. The authors have revised the original text to the following more precise statement:

“A study by Liu and colleagues from the Program in Cellular and Molecular Medicine at Boston Children’s Hospital revealed that DSF specifically *in vitro* inhibits the pore-formation of gasdermin D (GSDMD), a key mediator of pyroptosis.^12^ Although it remains unclear whether DSF directly inhibits GSDMD pore formation *in vivo*, and whether its anti-pyroptotic effect is mediated by DSF itself or its copper-metabolite, its antiseptic effect is significant.^12^”

These changes do not affect the results or conclusions of this review. The authors would like to apologise for any inconvenience caused.

The Royal Society of Chemistry apologises for these errors and any consequent inconvenience to authors and readers.

